# Widespread Molecular Patterns Associated with Drug Sensitivity in Breast Cancer Cell Lines, with Implications for Human Tumors

**DOI:** 10.1371/journal.pone.0071158

**Published:** 2013-12-27

**Authors:** Chad J. Creighton

**Affiliations:** Department of Medicine and Dan L. Duncan Cancer Center Division of Biostatistics, Baylor College of Medicine, Houston, Texas, United States of America; University of Porto, Portugal

## Abstract

**Background:**

Recent landmark studies have profiled cancer cell lines for molecular features, along with measuring the corresponding growth inhibitory effects for specific drug compounds. These data present a tool for determining which subsets of human cancer might be more responsive to particular drugs. To this end, the NCI-DREAM-sponsored DREAM7: Drug Sensitivity Prediction Challenge (sub-challenge 1) set out to predict the sensitivities of 18 breast cancer cell lines to 31 previously untested compounds, on the basis of molecular profiling data and a training subset of cell lines.

**Methods and Results:**

With 47 teams submitting blinded predictions, team Creighton scored third in terms of overall accuracy. Team Creighton's method was simple and straightforward, incorporated multiple expression data types (RNA-seq, gene array, RPPA), and incorporated all profiled features (not only the “best” predictive ones). As an extension of the approach, cell line data, from public datasets of expression profiling coupled with drug sensitivities (Barretina, Garnett, Heiser) were used to “predict” the drug sensitivities in human breast tumors (using data from The Cancer Genome Atlas). Drug sensitivity correlations within human breast tumors showed differences by expression-based subtype, with many associations in line with the expected (e.g. Lapatinib sensitivity in HER2-enriched cancers) and others inviting further study (e.g. relative resistance to PI3K inhibitors in basal-like cancers).

**Conclusions:**

Molecular patterns associated with drug sensitivity are widespread, with potentially hundreds of genes that could be incorporated into making predictions, as well as offering biological clues as to the mechanisms involved. Applying the cell line patterns to human tumor data may help generate hypotheses on what tumor subsets might be more responsive to therapies, where multiple cell line datasets representing various drugs may be used, in order to assess consistency of patterns.

## Introduction

Response to targeted therapy may vary from patient to patient, depending on the active pathways within the cancer being treated. These active pathways might be inferred, using the molecular profile of the cancer. As a step towards cataloguing molecular correlates of drug response, which might eventually yield markers for personalized therapy, recent studies have provided molecular profiling data (including gene expression and mutation) on large numbers of cancer cell lines (including ∼60 breast cancer cell lines), along with measurements of growth inhibitory effects for specific drug compounds [1], [2], [3]. These data represent a valuable resource for the possible development of molecular “signatures” that might eventually be used to predict drug response in patients.

While data are available for deriving candidate predictive signatures of therapeutic response, there are a multitude of ways in which the data may be analyzed. With the goal of identifying analysis methodologies that may be applied here, the NCI-DREAM consortium (DREAM standing for “Dialogue for Reverse Engineering Assessments and Methods”) recently sponsored a challenge (“sub-challenge 1” of the DREAM7: Drug Sensitivity Prediction Challenge), for research teams to use molecular data to predict the sensitivity of breast cancer cell lines to previously untested compounds. The Challenge participants submitted their blinded bioinformatics-based predictions, which were then evaluated empirically against the measured results, to see which algorithms had the best performance. As stipulated by the organizers, NCI-DREAM Challenge participants were invited as collaborators in the main NCI-DREAM consortium paper [4], which highlighted the top performing method, while providing high level descriptions of the methods used by the other teams.

The purpose of this paper is to describe in more detail, what ended being the third best performing method in the NCI-DREAM challenge (out of 47 submissions in all). The method was rather simple and straightforward in its approach, and did not make much effort to select the “best” predictive molecular features from the data, but rather weighted all available features according to their correlations with drug response. In this paper, we also explore the potential of using this method to “predict” drug response in human breast tumors, making use of data from The Cancer Genome Atlas (TCGA), by which clear distinctions based on tumor subtype could be observed.

## Results

### Basic approach

As part of the NCI-DREAM Challenge (“sub-challenge 1”), drug sensitivity measurements were made for 31 different drugs on 53 breast cancer cell lines. For 35 cell lines (the “training set”), the drug sensitivity values were made available, along with molecular data from a variety of platforms, including mRNA expression by both sequencing (RNA-seq) and gene array, protein expression by Reverse Phase Protein Arrays (RPPA), DNA methylation arrays, exome sequencing, and SNP arrays. For 18 cell lines (the “test set”), the drug sensitivity values were withheld from the Challenge participants. The identities of the drugs were also withheld until after submission.

**Figure 1:**
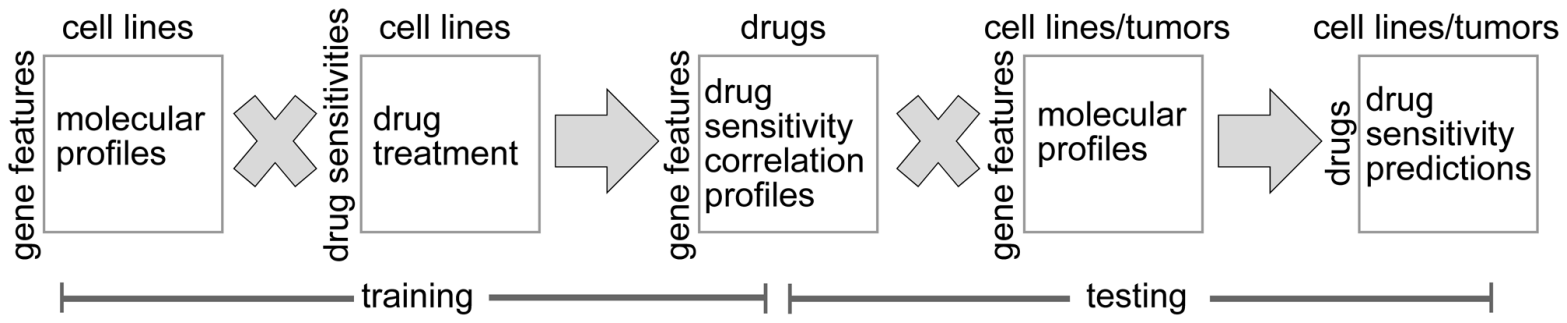
Schematic of the basic approach used to predict drug sensitivies. In the training phase of the analysis (using cell lines in the “training” set), the expression of each gene is correlated with the drug sensitivity measurements (e.g. GI50s). For the given drug, the associated molecular correlation profile of sensitivity is then correlated with each of the expression profiles from samples in the “test” set (e.g. from the NCI-DREAM test cell lines, or from human tumors in TCGA). The resulting prediction scores provide a relative measure for inferring drug sensitivity.

Figure 1 outlines the basic approach used by our NCI-DREAM Challenge Team #398 (Creighton), for predicting drug response based on molecular features. Of the molecular datasets provided for breast cancer cell lines, three were used: gene expression array, RNA-seq, and RPPA; the exome-seq and SNP array data were thought, perhaps, to be too sparse for the purposes of prediction, and DNA methylation data could optionally have been incorporated into our method but was not for the actual Challenge submission. Each dataset was first analyzed individually, in order to generate a set of predictions for the relative sensitivities across cell lines for a given drug; the resulting prediction scores from each platform were then averaged to obtain the final scores for submission.

The analytical method used was rather simple and straightforward, which to a degree may set it apart from other methods. For a given expression profile dataset, a matrix of correlations (by Pearson's) was constructed (across cell lines in the training set), between the drug sensitivities (−logged GI50 concentrations) and expression values. Within the matrix, each feature (e.g. gene or protein) would have a correlation value for each of the 31 drug compounds; a strong positive correlation would suggest that the feature might be a marker of sensitivity (e.g. *ERBB2*/*GRB7* for lapatinib −logGI50), and a strong negative correlation, a marker of resistance. In the prediction phase of the analysis, the Pearson's correlation was computed between each drug sensitivity profile (derived from the training cell lines) and each genomic profile (e.g. of gene/protein expression) of the test cell lines. A high correlation between drug sensitivity training profile and test sample genomic profile, would suggest that the test sample would be more sensitive to the drug (at least relatively speaking).

Notably, for each dataset, all features profiled were used in the scoring; in other words, there was no filtering or pre-selection for the “best” correlates or predictive features. Instead, all features were weighted by their correlation with drug senstivity, with features having little or no correlation pattern being weighted near zero. The assumption here was that the molecular patterns associated with drug sensitivity would be widespread, with perhaps on the order of hundreds of genes that could be used to drive the predictions.

### Gene classifier significantly predicts drug sensitivity in blinded test

Despite the simplicity of its approach, the prediction method of Team Creighton performed well in the Challenge. Out of 47 teams that submitted predictions, Team Creighton ranked third overall in terms of aggregated predicion accuracy. Using the global wac-index [4], Team Creighton had a score of 0.570 (*P* = 2.06E-05), while the Challenge winner, “teamfin”, had a score of 0.583 (with 0.5 being the expected score by chance). In addition to the aggregate score, the individual scores by compound could also be examined by team (Table 1). In addition to overall scoring, teamfin frequently performed well for individual drugs, while for a few drugs, team Creighton performed just slightly better over teamfin. Drugs where team Creighton performed notably less well, compared to teamfin, include Chloroquine, FTase inhibitor I, and Mebendazole.

**Table 1:** Concordance indices by drug for the top five performing teams.

	overall ranking	1	2	3	4	5		
ID	Drug name	teamfin	Team #632	Creighton	Team #433	Team #434	Best Team*	Difference, Creighton vs teamfin
1	Hydroperoxycyclophosphamide	0.584	0.545	0.553	0.622	0.612	Team #433	−0.03
2	Hydroperoxycyclophosphamide+Doxorubicin Combo	0.563	0.622	0.599	0.590	0.567	Team #632	0.04
3	Baicalein	0.604	0.544	0.499	0.555	0.539	teamfin	−0.10
4	Bromopyruvate	0.835	0.739	0.693	0.739	0.739	teamfin	−0.14
5	Cetuximab	0.599	0.518	0.522	0.579	0.620	Team #434	−0.08
6	Chloroquine	0.764	0.285	0.236	0.215	0.215	teamfin	−0.53
7	Disulfiram	0.486	0.585	0.480	0.289	0.304	Team #632	−0.01
8	Doxorubicin	0.579	0.607	0.608	0.454	0.499	Creighton	0.03
9	ERKi II (FR180304)	0.422	0.476	0.452	0.500	0.535	Team #434	0.03
10	Everolimus	0.693	0.651	0.615	0.614	0.594	teamfin	−0.08
11	FTase inhibitor I	0.700	0.569	0.372	0.370	0.338	teamfin	−0.33
12	GM6001	0.500	0.500	0.500	0.500	0.500	teamfin	0.00
13	GW5074	0.546	0.670	0.533	0.750	0.750	Team #433	−0.01
14	Herceptin	0.534	0.564	0.550	0.532	0.517	Team #632	0.02
15	IKK 16	0.578	0.649	0.647	0.718	0.720	Team #434	0.07
16	Imatinib	0.533	0.534	0.582	0.556	0.547	Creighton	0.05
17	Mebendazole	0.740	0.655	0.569	0.359	0.379	teamfin	−0.17
18	Methyl Glyoxol	0.579	0.647	0.573	0.415	0.377	Team #632	−0.01
19	MG-132	0.569	0.558	0.711	0.698	0.685	Creighton	0.14
20	Nelfinavir	0.502	0.583	0.577	0.521	0.517	Team #632	0.07
21	Nilotinib	0.532	0.548	0.452	0.524	0.524	Team #632	−0.08
22	Olomoucine II	0.398	0.415	0.537	0.587	0.580	Team #433	0.14
23	PD184352	0.725	0.673	0.737	0.711	0.711	Creighton	0.01
24	PS-1145	0.421	0.554	0.487	0.479	0.479	Team #632	0.07
25	QNZ	0.636	0.636	0.657	0.541	0.541	Creighton	0.02
26	Sodium Dichloroacetate	0.500	0.500	0.500	0.500	0.500	teamfin	0.00
27	TAPI-0 (IC-1)	0.500	0.500	0.500	0.500	0.500	teamfin	0.00
28	TCS PIM-11	0.467	0.548	0.598	0.788	0.780	Team #433	0.13
29	Valproic acid	0.535	0.465	0.467	0.608	0.619	Team #434	−0.07
30	Z-Leu-Leu-Leu-al	0.608	0.573	0.669	0.667	0.647	Creighton	0.06
31	Z-Leu-Leu-Norvalinal	0.701	0.653	0.672	0.720	0.660	Team #433	−0.03

Of the top five overall performing teams.

**Figure 2:**
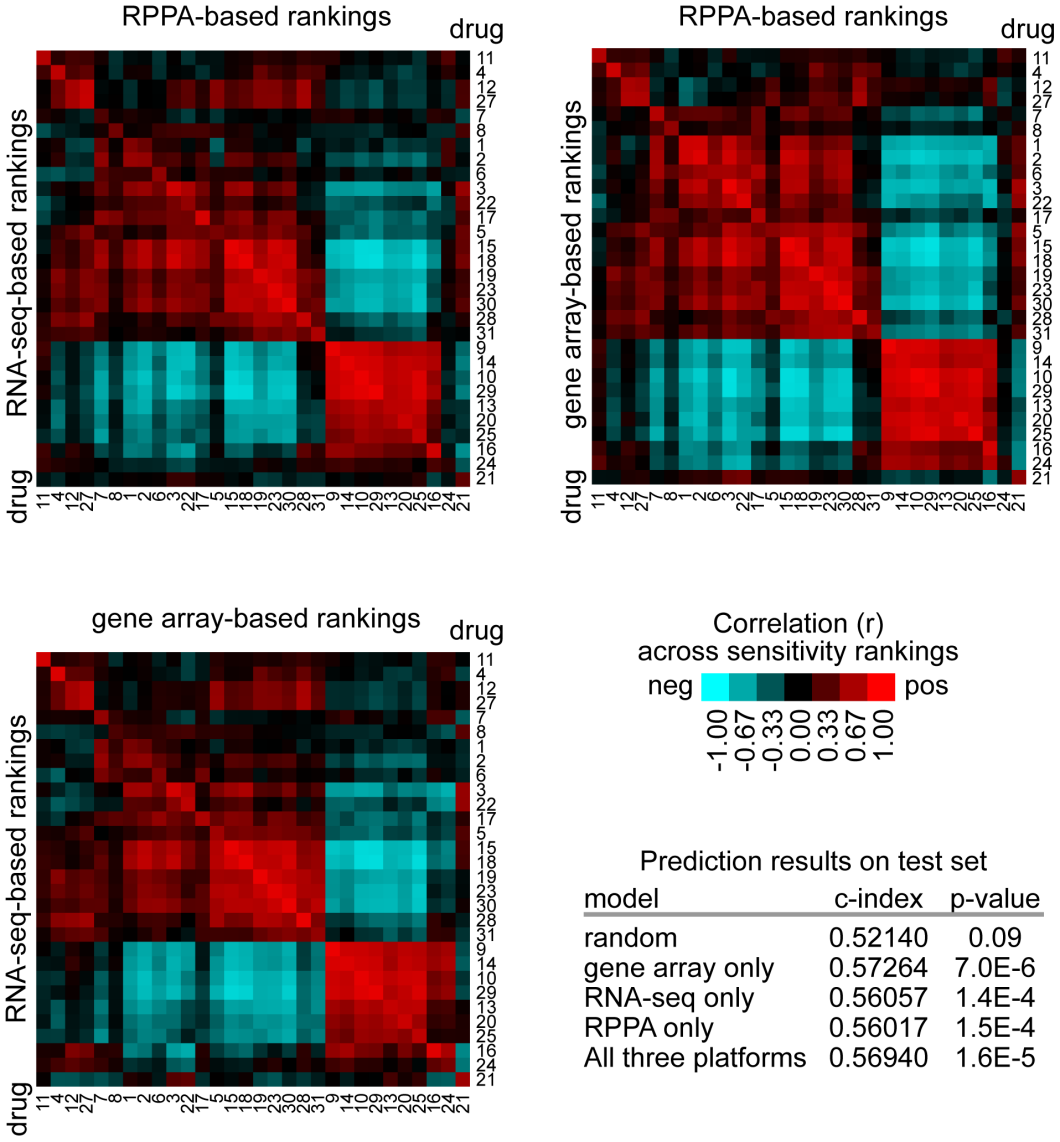
For the NCI-DREAM cell lines, drug response correlation patterns are concordant across different molecular platforms. For the Challenge submission by Team #398 (Creighton), the gene array, RNA-seq, and RPPA datasets were first analyzed separately, and the resulting prediction scores from each platform were then averaged to obtain the final scores for submission. Heat maps show correlations (by Spearman's) between drug sensitivity predictions for any two platforms (RNA-seq vs RPPA, gene array vs RPPA, RNA-seq vs gene array). Table shows overall prediction accuracy score (c-index), for predictions based on either an example random model (with many iterations expected to average around 0.5), gene array data alone, RNA-seq data alone, RPPA data alone, or the average of all three platforms. (The official Challege submission had training scores ordered by GI50, as prescribed by the Challenge organizers, while this figure has training scores ordered by molecular correlation-based predictions.)

By team Creighton's approach, predictions were made based on each individual molecular platform (gene array, RNA-seq, RPPA), then averaged for the final predictions. At the same time, the individual molecular platforms yielded predictions that were largely concordant with each other, and each platform-specific set of predictions performed similarly well as compared to chance (Figure 2). This suggests that, regarding drug sensitivity, each molecular data type examined contains potential information; at the same time, averaging the three sets of results might be helpful, in order to offset any relative deficiencies as observed in one platform by the relative strengths in another.

### Widespread molecular correlations with drug response in cell lines

Our successful prediction results would suggest that the molecular patterns underlying drug response may be widespread. While our actual Challenge submission used all available features, here we considered whether the predictive accuracies might have been improved, by using only those features having the strongest correlations (Table 2). Interestingly, based on analysis of the gene array dataset, predictive accuracies were just slightly worse (though still significant), when using the more restrictive feature sets. It would seem (at least using our method) that the potential improvement that might be had, by using only the top predictive features, could be outweighed by the detriment of losing information from the rest of the features that may miss an arbitrary statistical cutoff. In addition, the RPPA dataset in pariticular had only 131 protein features, and using rigorous cutpoints there would leave us few features for assessing correlation patterns.

**Table 2:** Evalutation of alternative cutoff points for defining top feature correlates (gene array dataset).

cutoff used	average features per drug	c-index	p-value
none	18632	0.57264	7.05E-06
P<0.05	1316	0.56718	2.89E-05
P<0.01	352	0.5605	0.000142
P<0.001	62	0.55496	0.000475

Cutoff based on Pearson's correlation between GI50 values and gene expression (training set).

Average features per drug do not include “Drug26.”

c-index, weighted average probablistic c-index, assessing predictive accuracy of gene-based scores.

**Figure 3:**
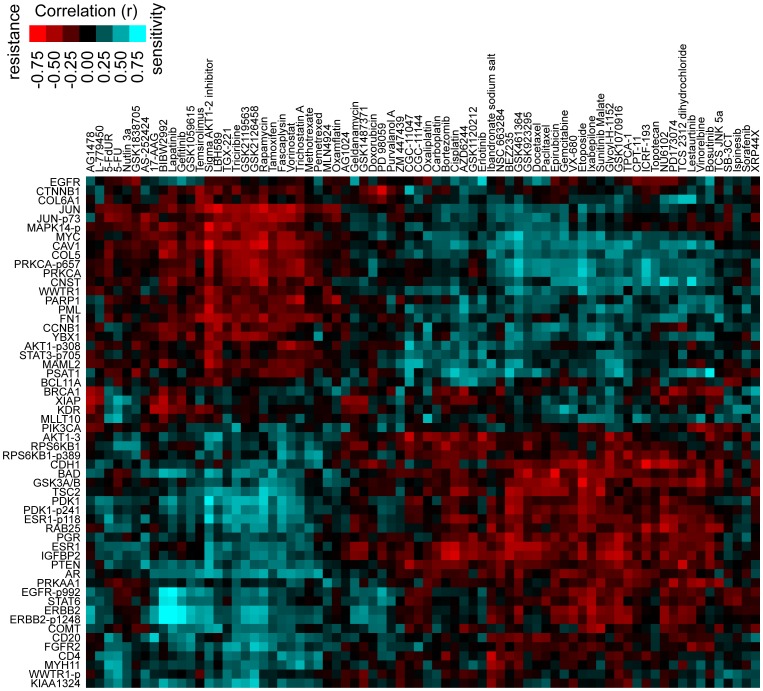
Global correlations between proteins and drug sensitivity measurement (using NCI-DREAM RPPA dataset and GI50 data from Heiser *et al.* [**2**]). The subset of protein represented here, are those having correlations with significance of P<0.01 for at least two compounds. Rows, proteins; columns, drugs. Blue, correlation with drug sensitivity; red, correlation with resistance.

In addition to its potential for prediction of drug response, the molecular data may also provide biological clues as to the mechanisms of drug response, in terms of the individual drug-to-feature correlations. For example, using the NCI-DREAM RPPA data, combined with the drug sensitivity measurements by Heiser *et al.* that corresponded to the cell lines [2], the top correlations between proteins and drug sensitivity could be visualized as a two-dimensional matrix (Figure 3). A clustering of the correlations indicated that there were at least two major groups of proteins and or drugs, in terms of overall similarity or dissimilarity of patterns; from what can be inferred from the markers and drugs involved, these groups should roughly coincide with the differences between estrogen receptor-positive (ER+) and ER− cell lines. The matrix visualization of marker-drug correlations highlighted a number of interesting associations (many of which could have been anticipated), one of the most prominent being that between expression of Her2 protein and sensitivity to HER2- inhibitors such as Lapatinib or BIBW2992. Also, EGFR expression correlated with sensitivity to Gefitinib and Erlotinib. ER and progesterone receptor (PR) proteins did correlate with Tamoxifen sensitivity, but interestingly, this was not one of the stronger correlations observed (perhaps due in part to the relative underrepresentation of ER+ versus ER− breast cancer cell lines).

### Drug sensitivity correlations within human breast tumors

Ultimately, we would like to be able to predict which subsets of human cancers may be responsive to therapy. In this sense, the “testing” phase of our basic approach (Figure 1) could involve data from human tumors as well as cell lines. To this end, breast cancer cell line data, from public datasets of expression profiling coupled with drug sensitivities—from Barretina *et al.* [1], Garnett *et al.* [3], and Heiser *et al.* [2]—were used as training data to “predict” the drug sensitivities in human breast tumors. The human data here were from The Cancer Genome Atlas [5], which provides comprehensive molecular profiles for over 500 tumors, at data levels that include mRNA, protein (by RPPA assay), microRNA, gene promoter methylation, DNA copy, and somatic mutation.

**Figure 4:**
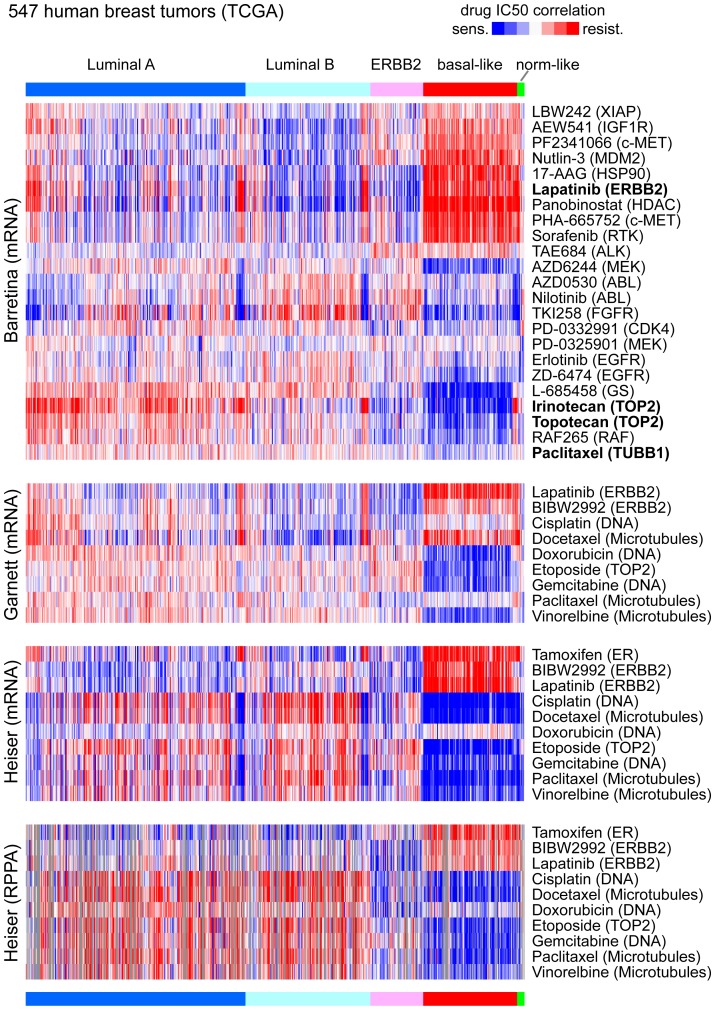
Drug sensitivity correlation patterns within human breast tumors. Three different datasets of cancer cell lines were examined (Barretina, Garnett, and Heiser), each dataset consisting of both molecular profiles and drug sensitivity measurements. For each cell line dataset, molecular correlation profiles of drug sensitivity were generated and applied to a separate profile dataset of human breast tumors (from TCGA). For each heat map, rows represent drugs, and columns represent human tumors (ordered by mRNA expression-based subtype); blue, correlation with drug sensitivity pattern; red, correlation with resistance pattern. For Barretina, all drugs with available data are shown, while for Garnett and Heiser, drugs shown include Tamoxifen, Her2 inhibitors, and chemotherapeutic agents represented in multiple studies. For Heiser, both mRNA data and RPPA data were used separately to generate correlation patterns (gray, no data).

For each drug represented in the cell line dataset, the correlations with gene or protein expression were projected onto the human tumor dataset, yielding a matrix of drugXtumor sensitivity correlations. These sensitivity correlations could be viewed as a heat map representation, for each cell line dataset that was used to derive the molecular feature correlates (Figure 4). For the Heiser dataset, both mRNA data and RPPA data were used separately to generate correlation patterns.

As might be expected, based on previous analyses of the cell line data [2], drug sensitivity correlations within human breast tumors showed clear differences by expression-based subtype (e.g. luminal A, luminal B, Her2-enriched, basal-like, and normal-like [5]). A number of these subtype-specific associations appeared to be in line with what might have been expected. For example, the Her2-enriched subtype showed sensitivity to anti-Her2 agents (e.g. Lapatinib and BIBW2992), the luminal subtype showed sensitivity to Tamoxifen, and the basal-like subtype showed sensitivity to chemotherapeutic agents [6]. Overall, the separate results sets using data from the three different cell line studies were concordant, though individual descrepancies could be seen as well (e.g. Docetaxol associations between Garnett and Heiser), which illustrates the utility of using multiple data sources in order to identify patterns of consensus.

### Basal-like tumors show patterns of resistance to PI3K inhibitors

**Figure 5:**
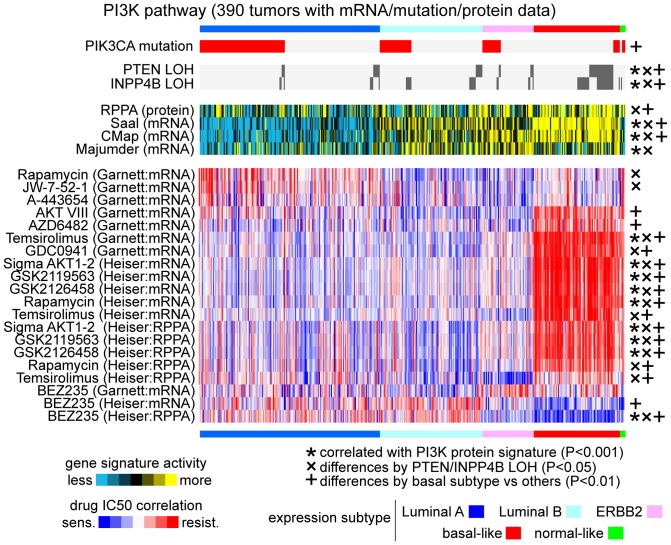
Basal-like tumors show patterns of resistance to PI3K inhibitors. Red-blue heat map represents drug sensitivity correlations within human breast tumors, for the subset of PI3K inhibitors (results were generated using either Garnett or Heiser datasets; red, correlation with resistance pattern). Also represented for these same tumors, are gene signature scores of inferred PI3K pathway activity (yellow: more active); four different PI3K activity signatures are represented, three of them based on mRNA patterns and one based on RPPA/proteomic patterns. *PIK3CA* somatic mutation and *PTEN* or *INPP4B* copy loss are also indicated. P-values by Pearson's correlation.

While many of the drug sensitivity associations, as observed in human breast tumors, could be verified using prior knowledge, other associations could invite further consideration and study. For example, when focusing on the subset of drugs that target the PI3K pathway, basal-like tumors, in general, showed patterns of resistance to PI3K inhibitors (Figure 5). The consensus pattern was evident when considering not just multiple independent datasets, but multiple drugs thought to target the same pathway. There were some notable exceptions to the overall pattern, such as the drug BEZ235 showing higher sensitivity in the basal-like group, as has previously been observed in the cell lines [2]. Interestingly, previous analyses of TCGA data, using gene and protein signatures of pathway activation, had indicated that the PI3K pathway was most active in the basal-like group. Genetic and genomic alterations in the PI3K pathway were previously found in all breast cancer subtypes, with basal-like cancers having more loss of pathway inhibitors *PTEN* and *INPP4B*, and with luminal A cancers having more mutations in *PIK3CA*.

## Discussion

We may conclude that cancer cells have a molecular signal indicative of potential drug response, based in part on the results of the NCI-DREAM Drug Sensitivity Prediction Challenge, where multiple analytical approaches, from various independent research teams, could yield significant accuracies of prediction. Notably, the Challenge used a rather small sample set, and even more robust patterns would likely emerge as more data on additional cell lines are considered. More data would also be needed, in order to assess whether the level of precision such molecular-based predictions can offer, would be useful for guiding treatment decisions for specific patients. One caveat of these types of analyses is that the predicted sensitivity rankings are relative, and in the case of patients, translating the predictions into recommended therapeutic doses may be difficult in some cases.

Given the good performance of our method in the Challenge results, we can give some consideration to the patterns associated with drug sensitivity in the human breast tumors. We find that these patterns segregate tumors by expression-based subtype, which should not be suprising, given the observed differences in drug sensitivities in the cell lines by their subtype [2], as well as the widespread molecular patterns that define the tumor subtypes [5]. These drug sensitivity-associated patterns may be helpful in generating hypotheses, of what patient subgroups might best respond to given therapy, though careful evaluation of all available data (including any clinical trials data) would be needed to better establish any associations.

Multiple cell line datasets representing various drugs that target similar pathway may be considered, in order to assess consistency of patterns as observed in human tumors. In particular, the observation, of basal-like breast tumors showing patterns of resistance to PI3K inhibitors, is intriguing, given that these tumors were found previously to show elevated PI3K signaling [5], [7]. One might expect, given our previous experiences with targeting ER and Her2, that those tumors having greater activity for the pathway would be more sensitive to therapies blocking that pathway; however, it is conceivable that not all pathways may follow this model of oncogene addition. Figuratively speaking, it may be, in some cases, that more water is needed to put out a bigger fire. More data, and more analysis and integration of the existing molecular datasets (from both cell lines and human tumors), are needed, regarding the overall question of drug response; however, this present study, coupled with the results of the NCI-DREAM Challenge, might be considered to represent a step forward.

## Materials and Methods

### Prediction of drug sensitivity in breast cancer cell lines

Prediction of drug sensitivity in breast cancer cell lines was carried out as part of the NCI-DREAM Drug Sensitivity Prediction Challenge [4], using the datasets provided by the Challenge organizers (including Growth Inhibitor, or GI, measurements, gene expression array, gene-level RNA-seq, and RPPA proteomic datasets). Methods are described elsewhere (NCI-DREAM manuscript in preparation). Briefly, for each dataset, features were first logged and centered on the median across samples. For a given dataset and the known GI values, a matrix of correlations (by Pearson's) was constructed (across cell lines in the training set) between negative logged GI concentration values and expression values. Using this matrix of [gene X drug] sensitivity correlations, the Pearson's correlation was then computed between each drug sensitivity profile and each genomic profile (e.g. of gene/protein expression) of the test cell lines, a high positive correlation for a given cell line denoting relatively higher drug sensitivity. Sensitivity predictions made using the three individual datasets were then averaged, in order to derive the final scores for submission. Example calculations in Excel, using the RPPA dataset, are available in Data S1. The “weighted average probablistic c-index,” used to assess the overall accuracy of a given set of predictions, is elsewhere described [4] and was computed here, using code provided by the Challenge organizers.

### Prediction of drug sensitivity in human breast tumors

Prediction of drug sensitivity in human tumors was carried out using public datasets [1], [2] [3] [5], using a very similar approach used for the Challenge submission as described above. The sets of tumors analyzed in TCGA cohort were the same as those analyzed previously as part of the initial data freeze [5], having either mRNA data or data on multiple platforms (mRNA, mutation, and RPPA data), as indicated. For TCGA datasets (gene array and RPPA), gene features were normalized across samples to standard deviations from median. Using a given cell line expression dataset (Barretina mRNA, Garnett mRNA, Heiser mRNA, NCI-DREAM RPPA), Pearson's correlation was computed between the expression of each gene and log (GI50 or IC50) across the cell lines; each TCGA breast tumor expression profile (either gene array or RPPA) was then correlated with each (cell line-derived) drug response profile. For Barretina and Garnett datasets, only the data from breast cancer cell lines were used in the analysis. For the “Heiser RPPA” correlations, the NCI-DREAM RPPA dataset was projected onto the Heiser drug sensitivity measurements. Java TreeView [8] represented matrix patterns as color maps. For PI3K pathway, transcriptomic and proteomic signatures of activity were previously applied to TCGA data [5], [9].

## Supporting Information

Data S1 **Calculations for predictions of drug response in cell lines, based on RPPA data (used for Challenge submission).**
(XLSX)
